# Food hygiene and safety measures among food handlers in street food shops and food establishments of Dessie town, Ethiopia: A community-based cross-sectional study

**DOI:** 10.1371/journal.pone.0196919

**Published:** 2018-05-03

**Authors:** Metadel Adane, Brhanu Teka, Yirga Gismu, Goitom Halefom, Muluneh Ademe

**Affiliations:** 1 Department of Environmental Health, College of Medicine and Health Sciences, Wollo University, Dessie, Ethiopia; 2 Department of Microbiology, Immunology and Parasitology, College of Health Sciences, Addis Ababa University, Addis Ababa, Ethiopia; 3 Department of Public health, College of Medicine and Health Sciences, Wollo University, Dessie, Ethiopia; Sciensano, BELGIUM

## Abstract

**Introduction:**

In sub-Saharan Africa foodborne disease and foodborne disease outbreaks are frequently ascribed to poor personal hygiene of street food vendors and food handlers in food establishments. Evidence on the level of food hygiene practices and food safety measures among food handlers is required for proper planning and implementation of targeted interventions. In this study, we aimed to determine the level of food hygiene and safety measures among street food vendors and food handlers in food establishments of Dessie Town, Ethiopia.

**Method:**

A community-based cross-sectional study was performed in Dessie town, Ethiopia from December 2013 to June 2014. Data were collected using a pre-tested structured questionnaire and an observational checklist by a trained data collector among 116 food handlers from 116 food establishments and 19 food handlers from 19 street food vendors. Multivariable logistic regression analysis with 95% confidence interval (CI) was used to identify the factors significantly associated with good level of food hygiene and safety practices.

**Result:**

Almost three-quarter (72%; 84/116) of food handlers in food establishments had a good level of food hygiene and safety practices compared to nearly half (53%; 10/19) of street food vendors. However, this difference was not statistically significant. Average monthly income of >$21 US (adjusted odds ratio [AOR] = 3.2; 95%CI: 1.3–7.7), availability of service training (AOR = 6.7; 95%CI: 1.8–25), wearing a gown during food handling (AOR = 19; 95%CI: 6.8–51) and medical checkup (AOR = 5.2; 95%CI: 2.1–13) were significantly associated with good levels of food hygiene and safety measures.

**Conclusion:**

Comprehensive health education and promotion programs through continuous training on food hygiene and safety, with promotion of wearing gown during food handling, regular medical checkups of food handlers and programs that enhance the monthly income of food handlers are promising strategies for promoting proper food handling practices in Dessie town, Ethiopia.

## Introduction

The World Health Organization (WHO) indicated that each year as many as 600 million people in the world fall ill of which 420,000 die after consuming contaminated food [1]. In the United States, foodborne illnesses affect an estimated 48 million people every year resulting in 128,000 hospitalizations and 3,000 deaths [2]. Africa and southeast Asia are believed to have the highest incidence and death rates associated to foodborne disease [1]. The repeated occurrence of foodborne disease has led to an increase in global concern about food hygiene and safety among food handlers [3]. However, epidemiological data on foodborne diseases remain scarce because cases often go unrecognized, unreported or uninvestigated [4]. In Ethiopia, there is a lack of documented information regarding the extent of foodborne diseases and the level of food hygiene and safety measures among street food vendors and food handlers of food establishments [5]. Foodborne disease outbreaks are often associated with poor personal hygiene of people handling foodstuffs. Food handlers therefore have significant role in ensuring food safety [6]. Thus, investigating the presence of knowledge gap on food hygiene and safety among street food vendors and food handlers of food establishments is a key step for proper planning and implementation of targeted interventions.

To our knowledge, no study has been undertaken on the level of food hygiene and safety among street food vendors and food handlers of food establishments in Dessie town, Ethiopia. Lack of such data hampers the design of effective intervention methods for improving food hygiene and safety among food handlers to prevent foodborne disease occurrences. A study by Aklilu et al. revealed that low hygiene and safety of food handlers are a potential risk of infection if sanitary conditions of food stuffs are not strictly followed [7]]. Hence, adherence to good personal hygiene and safe food handling practices remain an effective means of preventing foodborne disease transmission from food handlers to consumers [8, 9]. Furthermore, in Dessie town, dining out in food establishments and street shops is common.

Therefore, this study was designed to assess the status of food hygiene and safety measures among street food vendors and food handlers of food establishments of Dessie town, Ethiopia. The findings of this study may help urban health policy makers and program managers in the development and implementation of food hygiene and safety measures for preventing foodborne illness in Dessie town. Moreover, it could be taken as a benchmark to other similar towns in Ethiopia and throughout sub-Saharan Africa.

## Materials and methods

### Study area description

The study was conducted in Dessie town, a metropolitan town located in northern part of Ethiopia in Amhara National Regional State, South Wollo Zone at a distance of 401 km from the capital city Addis Ababa. Its astronomical location is 11°08’ latitude and 39°38’ longitude. Dessie town has 10 urban and 6 rural *kebeles* (the lowest administrative unit in Ethiopia consisting of at-least 5,000 population). Based on the population projection of Ethiopia, Dessie district in 2014 had a total population of 212,436 of which 84% (177,688/212,436) of them live in urban areas [10].

### Study design and period

This study was a community-based cross-sectional study, conducted from December 2013 to June 2014.

### Source and study population

The source populations were all food handlers in Dessie town who were working in food establishments and street food shops during the study period, whereas the study populations were those food handlers who were selected for the study using systematic sampling techniques.

### Inclusion and exclusion criteria

Food handlers who worked in food establishments and street food shops for at-least six months in Dessie town were included. Food establishments that had no food handlers during the data collection period were excluded.

### Sample size determination

The sample size was obtained using a double population proportion sample size estimation method [n = [Z_α/2_+Z_β_) ^2^ * (p_1_ (1-p_1_) + p_2_ (1-p_2_)] /(p_1_-p_2_)^2^] with an assumption of a proportion (P1) of medical checkup among food handlers of food establishments of 85% and a proportion (P2) of medical checkup among food handlers of street food shops of 64% with a power of 80% (Z_β_ = 0.84) and a 95% CI (Z_α/2_ = 1.96). Then, a 5% non-response rate was added. Finally, a total sample size for the two groups of 135 food handlers was determined. Since there is no equal number of food establishments and street food shops, we proportionally allocated the sample size to the two groups as described hereafter.

### Proportional allocation of sample size and sampling procedures

A preliminary survey was conducted in ten urban *kebeles* of Dessie town to identify the total number of food establishments and street food shops. There were 349 food establishments (hotel, restaurant, bar and restaurant, cafeteria and butcher houses) and 58 street food shops. A proportional allocation of sample size was performed for determining the number of study participants (food handlers) among food establishments and street food shops, i.e., 116 food handlers from 116 food establishments (135*349/407) and 19 food handlers from 19 street food shops (135*58/407). One food handler was considered from one food establishment and one street food shop.

The number of food handlers in street food shops was small compared to the number of food handlers in food establishments, due to the small number of existing street food shops. However, due to proportional allocation, it represents an equal allocation of the sample size among the two groups of food service provider. The selection of study participants was done in two steps. First, food establishments and street food shops were selected using systematic sampling technique with a sampling interval of three, which means that every third food establishment and every third street food shop was included. Then, one food handler was recruited from the selected food establishments and street food shops. When there was more than one food handler within food establishment or street food shop, one of them was randomly selected. When food handlers were not available from the selected food establishment or street food shop during the survey, we revisited once on the same day or the day after. Those who were not available during the revisits were considered as non-respondents.

### Data collection and data quality assurance

A pre-tested structured questionnaire and observational checklist were used to collect data from the study subjects. The structured questionnaire and observational checklist were first prepared in English and then translated into the local language, Amharic. The structured questionnaire was pretested on 10% of the sample size in one randomly selected nearby town to evaluate the face validity and to ensure whether food handlers of food establishments and street food vendors understood the questions. Based on the pre-test result, amendments were made for the questionnaire.

Four data collectors administered the pre-tested questionnaire by interviewing food handlers. The principal investigator provided two days of training for field data enumerators about the administration of each question and ethical principles. Daily supervision was done by the principal and co-investigators to check the completeness and reliability of the data. Collected data were entered in SPSS version 24.0 and then cleaned before analysis. In order to verify the accuracy of data entries, two generic data verification strategies were employed. As the first step, a random selection of 10% of the questionnaires was thoroughly checked. Then, descriptive statistics, including results from cross-tabulations and frequency distributions were examined before performing statistical analysis.

### Data analysis

Data were analyzed using SPSS version 24.0. Proportions for categorical variables and mean ± SD (standard deviations) for continuous variables were used as descriptive measures. Food handlers’ level on food hygiene and safety measures was set using 14 questions. For the descriptive statistics of level of food hygiene and safety, three categories were considered, i.e., poor: ≤60%, good: 60%–80%, and very good: 80%–100% [11]. For further analyses, food handlers who scored ≥9 points were considered to have a good level of food hygiene and safety practice, whereas food handlers who scored <9 points were considered to have a poor level of food hygiene and safety. Pearson chi square test was performed to compare the level of food hygiene and safety between street food vendors and food handlers in food establishments.

Bivariate (Crude odds ratio (COR)) and multivariable (adjusted odds ratio (AOR)) logistic regressions with corresponding 95%CI were used to assess the strength of associations with different variables. Variables with p-value < 0.2 in the bivariate analysis were considered for initial inclusion in the multivariable analysis. Hosmer-Lemeshow statistics were used to test the goodness-of-fit of the model. From the multivariable analysis, variables with p-value < 0.05 in the final model were taken as statistically significant and independently associated with a good level of food hygiene and safety measures among food handlers.

### Ethical consideration

Ethical clearance was obtained from the Institutional Ethical Review Committee of Wollo University, College of Medicine and Health Sciences. The Committee provided ethical approval after reviewing both the protocol and consent forms. Before data collection, permission was taken from owners of food establishments and written informed consent was obtained from study participants. Confidentiality was insured by collecting the data anonymously and coding the names of the respondents.

## Result

### Characteristics of the study participants

The response rate of the study participants was 100%. All food handlers in street food shops and the majority of food handlers in food establishments (80%; 93/116) were women. About less than half (41%; 56/135) of the study participants were grade 9–12 and about two-thirds (64%; 87/135) were single in marital status (Table 1). The mean age street food vendors and food handlers of food establishments was 24±9.6 years and 25±8.7 years, respectively.

**Table 1:** Socio-demographic characteristics of street food vendors and food handlers in food establishments from December 2013 to June 2014, Dessie town, Ethiopia.

Socio-demographic Variables	Street food vendors (n = 19)	Food handlers in food establishments (n = 116)	Total
Sex			
Women	19 (100%)	93 (80%)	112 (83%)
Men	0 (0%)	23 (20%)	23 (17%)
Age			
<18 years	4 (21%)	6 (5.2%)	10 (7.4%)
≥18 years	15 (79%)	110 (95%)	125 (93%)
Marital status			
Single	11 (58%)	76 (66%)	87 (64%)
Married	6 (32%)	27 (23%)	33 (24%)
Divorced	2 (10%)	12 (10%)	14 (10%)
Widowed	0 (0%)	1 (0.9%)	1 (0.7%)
Religion			
Orthodox	9 (47%)	75 (65%)	84 (62%)
Muslim	10 (53%)	35 (30%)	45 (33%)
Protestant	0 (0%)	4 (3.4%)	4 (3.1%)
Others	0 (0%)	2 (1.7%)	2 (1.5%)
Educational status			
Illiterate	2 (11%)	15 (13%)	17 (13%)
Grade 1–4	1 (5.3%)	6 (5.2%)	7 (5.2%)
Grade 5–8	8 (42%)	34 (29%)	42 (31%)
Grade 9–12	6 (32%)	50 (43%)	56 (41%)
College/above	2 (10%)	11 (9.5%)	13 (9.6%)
Average monthly income			
≤21 US$	18 (95%)	61 (53%)	79 (58%)
>21 US$	1 (5.3%)	55 (47%)	56 (42%)

### Overall compliance score of the level of food hygiene and safety measures

Our results showed that food handlers in food establishments had a better level of compliance score for food hygiene and safety measures (9.8±2.2) compared to food handlers in street food shops (8.9±2.0). The overall compliance score was “very good” for 26% (30/116) of food handlers in food establishments versus 16% (3/19) of food handlers in street food shops. Moreover, the overall compliance score was “poor” for 28% (32/116) food handlers in food establishments versus 47% (9/19) food handlers in street food shops (Table 2).

**Table 2:** Compliance score of the level of food hygiene and safety measures among street food vendors and food handlers in food establishments from December 2013 to June 2014, Dessie town, Ethiopia.

Level of food hygiene and safety measure	Street food vendors (n = 19)	Food handlers in food establishments (n = 116)
Mean score ±SD	8.9±2.0	9.8±2.2
**Level (Score)**		
Poor (0–8)	9 (47%)	32 (28%)
Good (9–11)	7 (37%)	54 (46%)
Very good (12–14)	3 (16%)	30 (26%)

SD = Standard deviation

### Comparison of level of food hygiene and safety measures

Nearly three-quarters of food handlers in food establishments (72%; 84/116) had good level of food hygiene and safety as compared to food handlers in street food shops (53%; 10/19) (Table 2). However, the difference in the level of food hygiene and safety measures among the two groups was not statistically significant (p = 0.210). Comparison of the measures of food hygiene and safety measures among street food vendors and food handlers in food establishments is summarized in Table 3. Furthermore, the levels of food hygiene and safety measures are summarized in Table 4.

**Table 3:** Comparison of measures of food hygiene and safety among street food vendors and food handlers in food establishments from December 2013 to June 2014, Dessie town, Ethiopia.

Variables	SFV* (n = 19)	FE* (n = 116)	Total
Awareness on fecal-oral disease transmission			
Yes	19 (100%)	116 (100%)	135 (100%)
Can you mention foodborne illness			
Yes	17 (89%)	111(96%)	128 (95%)
No	2 (11%)	5 (4.3%)	7 (5.2%)
Do you use separate handmade (fabric) towel for dish drying			
Yes	14 (74%)	65(56%)	79 (58%)
No	5 (26%)	51 (44%)	56 (42%)
Will dish drying towel be a contamination risk			
Yes	1 (7.1%)	19 (29%)	20 (25%)
No	13 (93%)	46 (71%)	59 (75%)
Do you know the importance of gowning			
Yes	15 (79%)	106 (91%)	121 (90%)
No	4 (21%)	10 (8.6%)	14 (10%)
If yes, Gown is used for			
Identification ID	3 (20%)	32 (30%)	35 (29%)
Hygiene	7 (47%)	53 (50%)	60 (50%)
Both hygiene & ID	5 (33%)	21 (20%)	26 (21%)
Usually wear gown at work			
Yes	6 (32%)	78 (67%)	84 (62%)
No	13 (68%)	38 (33%)	51 (38%)
Wear gown at study visit (observed)			
Yes	7 (37%)	81 (70%)	88 (65%)
No	12 (63%)	35 (30%)	47 (35%)
Do you wash your hand after latrine			
Yes	19 (100%)	111 (96%)	130 (96%)
No	0 (0%)	5 (4.3%)	5 (3.7%)
Does washing hand after latrine prevent foodborne disease			
Yes	19 (100%)	111 (96%)	130 (96%)
No	0 (0%)	5 (4.3%)	5 (3.7%)
Have you taken a training related to food handling			
Yes	5 (26%)	35 (30%)	40 (30%)
No	14 (74%)	81 (70%)	95 (70%)
Medical checkup within six months			
Yes	13 (68%)	83 (72%)	96 (71%)
No	6 (32%)	33 (28%)	39 (29%)
If yes, do you have checkup certificate			
Yes	4 (31%)	41 (49%)	45 (47%)
No	9 (69%)	42 (51%)	51 (53%)
Is medical checkup periodic			
Yes (quarterly)	8 (62%)	55 (66%)	63 (66%)
Yes (6–12 months)	3 (23%)	16 (19%)	19 (20%)
Not periodic	2 (15%)	12 (15%)	14 (14%)

*SFV = Street food vendors; FE = Food handlers in food establishments

**Table 4:** Bivariate analysis of the level of food hygiene and safety measures among all food handlers from December 2013 to June 2014, Dessie town, Ethiopia.

Variables (n = 135)	Level of food hygiene and safety measures		
	Good	Poor	COR (95%CI)
Sex			
Women	75 (67%)	37 (33%)	0.4 (0.14, 1.34)
Men	19 (83%)	4 (17%)	1.0
Age			
<18 years	3 (30%)	7 (70%)	0.16 (0.04, 0.66)
≥18 years	91 (73%)	34 (27%)	1.0
Marital status			
Single	58 (67%)	29 (33%)	1 (0.31, 3.2)
Married	26 (79%)	7 (21%)	1.9 (0.48, 7.23)
Divorced and widowed	10 (67%)	5 (33%)	1.0
Religion			
Christian	69 (76.7%)	21 (23.3%)	2.6 (1.22, 5.65)
Muslim	25 (55.5%)	20 (45.5%)	1.0
Educational status			
Illiterate	14 (82)	3 (18%)	1.4 (0.23, 8.42)
Grade 1–4	5 (71%)	2 (29%)	0.7 (0.09, 6.04)
Grade 5–8	28 (67%)	14 (33%)	0.6 (0.14, 2.53)
Grade 9–12	37 (77%)	19 (33%)	0.6 (0.14, 2.38)
College and above	10 (77%)	3 (23%)	1.0
Average monthly income			
>$21 US	47 (59%)	9 (16%)	3.6 (1.53–8.26)
≤$21 US	47 (84%)	32 (41%)	1.0
*Awareness on fecal-oral disease transmission	94 (70%)	41 (30%)	
*Can you mention foodborne illness			
Yes	93 (73%)	35 (27%)	15.9 (1.85, 137.19)
No	1 (14%)	6 (86%)	1.0
*Do you use separate handmade (fabric) towel for dish drying			
Yes	63 (80%)	16 (20%)	3.2 (1.48, 6.79)
No	31 (55%)	25 (45%)	1.0
*Will dish drying towel be a contamination risk			
Yes	19 (95%)	1 (5.0%)	10.1 (1.31, 78.49)
No	75 (65%)	40 (35%)	1.0
*Do you know the importance of gowning			
Yes	91 (75%)	30 (25%)	10.8 (2.82, 41.11)
No	3 (21%)	11 (79%)	1.0
*Usually wear gown at work			
Yes	76 (90%)	8 (10%)	17.4 (6.89, 44.04)
No	18 (35%)	33 (65%)	1.0
*Wear gown at study visit (observed)			
Yes	79 (90%)	9 (10%)	18.7 (7.44, 47.12)
No	15 (32%)	32 (68%)	1.0
*Do you wash your hand after latrine			
Yes	93 (72%)	37 (28%)	10.1 (1.09, 92.96)
No	1 (20%)	4 (80%)	1.0
*Do you use soap for hand washing			
Yes	91 (72%)	36 (28%)	4.2 (0.96, 18.55)
No	3 (38%)	5 (62%)	1.0
*Does washing hand after latrine prevent foodborne disease			
Yes	92 (71%)	38 (29%)	3.6 (0.58, 22.61)
No	2 (40%)	3 (60%)	1.0
*Have you taken a training on food handling			
Yes	37 (92%)	3 (8.0%)	8.2 (2.36, 28.59)
No	57 (60%)	38 (40%)	1.0
*Medical checkup within six months			
Yes	77 (80%)	19 (20%)	5.2 (2.34, 11.76)
No	17 (44%)	22 (56%)	1.0
*If yes, do you have checkup certificate			
Yes	43 (96%)	2 (4.4%)	16.4 (3.75, 72.07)
No	51 (57%)	39 (43%)	1.0
*Is medical checkup periodic			
Yes (quarterly)	58 (92%)	5 (7.9%)	15.5 (3.82, 62.61)
Yes (6–12 months)	12 (63%)	7 (37%)	2.3 (0.56, 9.37)
Not periodic	6 (43%)	8 (57%)	1.0

* Variables used to measure the level of food hygiene and safety

All food handlers in this study had awareness about fecal-oral disease transmission. The majority of street food vendors (89%; 17/19) and food handlers in food establishments (96%; 111/116) mentioned at least one illness linked to consumption of contaminated food and drink of which typhoid fever and typhus were repeatedly indicated (Table 5).

**Table 5:** Foodborne illnesses reported by food handlers in street food shops and food establishments from December 2013 to June 2014, Dessie town, Ethiopia.

No.	Foodborne illnesses	Number (n = 135)*	Percentage (%)
1	Typhoid fever	128	95
2	Typhus	96	71
3	Diarrhea	27	20
4	Amoebiasis	25	19
5	Vomiting	18	13
6	Stomachache	11	8.1
7	Giardiasis	7	5.2

* For each illness, more than one foodborne illnesses may be reported in one food handlers

Handmade or fabric towels were used for drying dishes by 59% (79/135) of study participants. Among food handlers in food establishments who used handmade or fabric towels, only 29% (19/65) of them had awareness whether towel could be the contamination risk. Seventy nine percent of street food vendors (15/19) and 91% (106/116) of food handlers in food establishments used to know the importance of wearing gown. However, only 37% (7/19) of street food vendors and 70% (81/116) of food handlers in food establishments appeared to wear gown during the study visit (Table 3). Among the food handlers who wore gown during the study visit, 90% (79/88) of them had a good level of food hygiene and safety.

About 72% (83/116) of food handlers in food establishments and 68% (13/19) of street food vendors had received a medical checkup within six months before this study. In 85% (82/96) of the cases, medical checkups were given at regular basis (quarterly or annually). Nearly a quarter of street food vendors (26%; 5/19) and 30% (35/116) of food handlers in food establishments received service training regarding food preparation and handling (Table 3). Among the food handlers who had received service training, 93% (37/40) of them had a good level of food hygiene and safety measures (Table 4).

### Factors associated with good level of food hygiene and safety measures in multivariable logistic regression analysis

From the multivariable logistic regression analysis, our findings indicated that average monthly income of above $21 US, availability of service training, medical checkup and wearing a gown during food handling were significantly associated with good level of food hygiene and safety practices. The odds of having good food hygiene and safety practices were 5.2 times higher (AOR = 5.2; 95%CI, 2.1–13) among food handlers that had received a medical checkup than food handlers who had not received medical checkup. We also found that the odds of having food hygiene and safety practices were 6.7 times higher (AOR = 6.7; 95%CI: 1.8–25) among food handlers that had received service training on food preparation and handling than food handlers that had no training opportunity. Moreover, food handlers with average monthly income of >$21 US had 3.2 times more likely (AOR = 3.2; 95%CI: 1.3–7.7) to have good food hygiene and safety practices as compared to those with a lower monthly income (Table 6).

**Table 6:** Factors significantly associated with good level of food hygiene and safety measures among street food vendors and food handlers in food establishments in multivariable logistic regression analysis from December 2013 to June 2014, Dessie town, Ethiopia.

Variables	Level on food hygiene and safety measures			
	Good	Poor	COR (95% CI)	AOR (95% CI)
Medical checkup				
Yes	77	19	5.2 (2.34–11.76)	5.2 (2.13–12.69)
No	17	22	1.00	1.00
Training				
Yes	37	3	8.2 (2.37–28.59)	6.7 (1.81–24.84)
No	57	38	1.00	1.00
Average monthly income				
>$21 US	47	9	3.6 (1.53–8.26)	3.2 (1.34–7.71)
≤$21 US	47	32	1.00	1.00
Wear gown on the visit				
Yes	79	9	18.7 (7.44–47.12)	18.6 (6.82–50.77)
No	15	32	1.00	1.00

## Discussion

We conducted a community-based cross-sectional study among street food vendors and food handlers in food establishments in Dessie town, Ethiopia. Our main findings showed that almost three-fourth (72%; 84/116) of food handlers in food establishments and about half (53%; 10/19) of street food vendors had a good food hygiene and safety practices. However, this difference was not statistically significant. We also found that average monthly income of above $21 US, availability of service training, wearing a gown during food handling and medical checkup were significantly associated with good levels of food hygiene and safety practices.

Our findings showed a better level of food hygiene and safety practices (72%) than previous studies conducted in other Ethiopian towns, i.e., Mekelle (64%) [12] and Dangila (53%) [13]. These differences might be a result of variation of socio-demographic factors among food handlers. Moreover, the studies were conducted in different periods and the findings might indicate year by year improvements in good levels of food hygiene and safety practices among food handlers. Our finding on good levels of food hygiene and safety practices among food handlers working in street food shops (53%) was comparable with a similar study in Gondar (58%), Ethiopia [14] but was lower than another study in Ghana (67%) [15].

We also found that the majority of food handlers in street food shops were below 18 years old. This finding is consistent with a study in Jimma, Ethiopia [16] in which street vending was dominated by youths. This might be due to low educational status and work experience of teenagers which might be below the entry requirements of food establishments.

Our study also showed that food handlers of a street food shop were less likely to wear a gown during food handling than food handlers in food establishments. This finding is in consistent with studies in Kenya [17] and Ghana [18]. This might be due to negligence or less pressure to wearing a gown compared to food handlers in food establishments where there might be a dress code.

Our finding also revealed that service training was one of the determinants for developing a good level of food hygiene and safety practices. This finding is consistent with studies conducted in Benin [19], Nigeria [20] and a study in another part of Ethiopia [21]. In contrast with our findings, however, a study in Korea [22] indicated that there is no significant difference in practices and sanitation performance of food handlers after training. This might be due to the differences in the type and method of training delivery or due to inconsistent and interrupted training programs.

Furthermore, we found that food handlers who had a better monthly income had a good chance of developing good levels of food hygiene and safety practice. The higher income might help to buy those conditions that improve hygiene and safety measures. Likewise, a well-paid food handler may have a sense of responsibility and commitment to adhere to measures of food hygiene and safety. A study by Hoque showed that a higher household income was associated with good hand washing behavior which promotes hygiene [23]. Consistent with our findings, a study in Addis Ababa showed that women who had higher income more likely sought healthcare compared to those who had a lower income [24]. Furthermore, another study in Dangila, Ethiopia showed that good food handling practices were practiced by food handlers with a higher monthly income [13].

### Limitations of the study and opportunities for future research

Our study was based on self-reporting, and the results were therefore prone to social desirability bias. Further studies using mixed methods of both quantitative and qualitative approach may bring a more comprehensive result and validate our finding. We also encourage further studies that explore the practice of hand washing with soap at critical times by the food handlers of food establishments and street food shops. Further studies may also differentiate food handlers that work in the kitchen and prepare food from food handlers that provide service after the preparation of food, in order to support the development of targeted interventions.

## Conclusion

We found an almost similar level of food hygiene and safety practices among food handlers of food establishments and street food shops. Service training, medical checkup, wearing a gown during food handling and average monthly income were factors significantly associated with good levels of food hygiene and safety by food handlers. Based on the findings of the study, comprehensive health education and promotion programs through continuous training about food hygiene and safety, with promotion of gown wearing during food handling; regular medical checkups of food handlers; and programs that enhance the monthly income of food handlers are promising strategies for promoting proper food handling practices in Dessie town, Ethiopia. To achieve this goal, the local government officials, non-governmental organizations (NGOs) and civic societies should work coordinately towards effective implementation of food hygiene and safety measures among food handlers of food establishments and street food vendors.
